# The Trend of Clinical Profile of Patients with Alcohol Dependence and Dual Diagnosis. A 10 Year Retrospective Comparative Study from a Tertiary Care Alcohol Addiction Service Centre in South India

**DOI:** 10.1192/bjo.2025.10219

**Published:** 2025-06-20

**Authors:** Sreeja Sahadevan, Indu Suresh, Kalesh M Karun

**Affiliations:** 1 Norfolk and Suffolk Foundation NHS Trust, Norwich, United Kingdom; 2 Kabani Wellness Centre, Kerala, India; 3 ICMR-National Institute of Traditional Medicine, Belagavi, India

## Abstract

**Aims:** Dual diagnosis in the context of alcohol dependence refers to the co-occurrence of alcohol dependence syndrome and another mental health condition, such as depression, anxiety, bipolar disorder, schizophrenia, or personality disorders. Understanding the clinical profile of patients with dual diagnosis can lead to more effective treatment and outcome. This retrospective study compares the trend and associated factors of clinical profile of people with alcohol dependence with and without dual diagnosis.

**Methods:** This is a retrospective medical records review conducted in a tertiary care de-addiction centre in South India. We reviewed the data of 2105 patients admitted to the centre with a diagnosis of alcohol dependence syndrome over 10 years from April 2007 to March 2017. Socio-demographic data, alcohol drinking pattern, clinical profile and dual diagnosis were extracted from the patient intake proforma and case records. Statistical analysis was done using R software. Trend of clinical profile was visualized by line diagram as dependent variable (y), years of study as independent variable (x) and analysis was done using linear regression. Chi square and Fisher exact tests were applied as univariate analysis and logical regression for multivariate analysis.

**Results:** Out of 2105 patients, all were males, with mean age 36 years (SD=9). Among them 1566 (74%) had a dual diagnosis of Bipolar disorder [974 (62%)], depression [323 (20%)], psychotic disorders [162 (10%)], antisocial personality disorder [107 (7%)] and others [16 (1%)]. Trend analysis revealed that the proportion and pattern of dual diagnosis and socio-demographic and clinical profile remained the same over years. In univariate analysis factors like binge drinking (p=0.002), domestic violence (p=0.04), occupational dysfunction (p=0.038) and debts (p=0.029) had significance in patients with dual diagnosis. However multivariate analysis did not support the same.

**Conclusion:** Comparing the existing literature, proportion of dual diagnosis is higher in our study, mood disorders being the commonest. Though there are changes in social conditions over the decade the profile of patients seeking de-addiction remained almost same. The social complications of alcohol were more pronounced in the dual diagnosis group. The findings can help train healthcare professionals to better understand and manage dual diagnosis cases. Dual diagnosis is not only a diagnostic challenge, but it should alert the treatment team regarding the pharmacological and psychosocial management as well. Research on dual diagnosis and alcohol dependence is vital for improving patient outcomes, reducing societal burdens, and advancing scientific understanding.

